# Clinical and physiological effects of transcranial electrical stimulation position on motor evoked potentials in scoliosis surgery

**DOI:** 10.1186/1748-7161-5-3

**Published:** 2010-02-23

**Authors:** YL Lo, YF Dan, YE Tan, A Teo, SB Tan, WM Yue, CM Guo, S Fook-Chong

**Affiliations:** 1 Department of Neurology, National Neuroscience Institute, Singapore General Hospital, Outram Road, Singapore 169608, Singapore; 2 Department of Neurology, Singapore General Hospital, Outram Road, Singapore 169608, Singapore; 3 Department of Orthopedic Surgery, Singapore General Hospital, Outram Road, Singapore 169608, Singapore; 4 Department of Clinical Research, Singapore General Hospital, Outram Road, Singapore 169608, Singapore

## Abstract

**Background:**

During intraoperative monitoring for scoliosis surgery, we have previously elicited ipsilateral and contralateral motor evoked potentials (MEP) with cross scalp stimulation. Ipsilateral MEPs, which may have comprised summation of early ipsilaterally conducted components and transcallosally or deep white matter stimulated components, can show larger amplitudes than those derived purely from contralateral motor cortex stimulation. We tested this hypothesis using two stimulating positions. We compared intraoperative MEPs in 14 neurologically normal subjects undergoing scoliosis surgery using total intravenous anesthetic regimens.

**Methods:**

Trancranial electrical stimulation was applied with both cross scalp (C3C4 or C4C3) or midline (C3Cz or C4Cz) positions. The latter was assumed to be more focal and result in little transcallosal/deep white matter stimulation. A train of 5 square wave stimuli 0.5 ms in duration at up to 200 mA was delivered with 4 ms (250 Hz) interstimulus intervals. Averaged supramaximal MEPs were obtained from the tibialis anterior bilaterally.

**Results:**

The cross scalp stimulating position resulted in supramaximal MEPs that were of significantly higher amplitude, shorter latency and required lower stimulating intensity to elicit overall (Wilcoxon Signed Rank test, p < 0.05 for all), as compared to the midline stimulating position. However, no significant differences were found for all 3 parameters comparing ipsilaterally and contralaterally recorded MEPs (p > 0.05 for all), seen for both stimulating positions individually.

**Conclusions:**

Our findings suggest that cross scalp stimulation resulted in MEPs obtained ipsilaterally and contralaterally which may be contributed to by summation of ipsilateral and simultaneous transcallosally or deep white matter conducted stimulation of the opposite motor cortex. Use of this stimulating position is advocated to elicit MEPs under operative circumstances where anesthetic agents may cause suppression of cortical and spinal excitability. Although less focal in nature, cross scalp stimulation would be most suitable for infratentorial or spinal surgery, in contrast to supratentorial neurosurgical procedures.

## Background

Intraoperative monitoring (IOM) of the motor pathways is a routine procedure for ensuring integrity of corticospinal tracts during scoliosis surgery. We have documented in previous studies the methodology and applications of bilaterally recorded motor evoked potentials (MEP), incorporating the monitoring of MEPs ipsilateral and contralateral to the active stimulating electrode. Our findings have suggested that bilateral motor cortex stimulation has resulted in ipsilateral MEPs (ipsilateral to the side of active cortical stimulating electrode), which may have comprised early ipsilaterally conducted components and late transcallosally stimulated components.

In practice, MEPs are usually with the C3C4 cross scalp or C3Cz/C4Cz midline stimulating positions during IOM. To our knowledge, there are no reported instances of false negatives with neurological deficits reported in the literature in relation to either stimulating position during scoliosis surgery. However, obtaining concurrent ipsilateral and contralateral MEP amplitude are of value in reducing false positive observations during IOM [1,2]. To this end, it would therefore be of interest to ensure that large, distinct and reproducible MEPs can be obtained ipsilaterally and contralaterally during surgery. Here, we aim to compare the efficacy of eliciting MEPs from 2 stimulating positions, clarify the predominant underlying mechanisms involved, as well as address any arising practical implications.

## Methods

Over a 1-year period, IOM was performed for 14 patients (13 females; mean age: 17; age range: 14 to 23) with idiopathic scoliosis. All were asymptomatic, neurologically normal and underwent correction for thoracic level scoliosis. The patients underwent total intravenous anesthesia (TIVA), maintained with propofol infusion. The institution's ethics committee had previously approved the study protocols.

Stimulation output was increased in steps of 5 mA until a morphologically reproducible MEP with the largest amplitude was elicited. The intensity was then increased and fixed at 10% above this threshold intensity to obtain a supramaximal MEP response (termed maximum MEP response). MEP recordings were obtained with 13 mm disposable subdermal needles (Technomed Europe, Beek, Netherlands) in the tibialis anterior (TA) bilaterally. Filter settings were set at 10 Hz and 2 kHz. Input impedance of stimulating and recording electrodes were maintained below 5 kOhm.

Stimulating electrodes consisted of 9 mm gold-plated disc electrodes at C3C4 (International 10-20 system) affixed with collodion. C3 was the active stimulating electrode position for left cortical stimulation, while C4 was for right cortical stimulation correspondingly. This was termed cross scalp stimulating position. The midline stimulating position consisted of electrodes C3 (left cortex stimulation) or C4 (right cortex stimulation) referenced to the midline at Cz.

For induction of anesthesia, sodium thiopentone at 4 mg/kg and fentanyl at 2 mcg/kg was administered. 0.8 mg/kg of intravenous atracurium was used to facilitate endotracheal intubation. No further doses of neuromuscular blocking agents were used subsequently. For TIVA, anesthesia was maintained using the regimen of 10 mg/kg of propofol for the first 10 minutes, 8 mg/kg for the nest 10 minutes and 5 mg/kg for the subsequent length of operation. 50% air in oxygen was administered. Morphine was titrated as required for pain relief. Monitoring included electrocardiography, pulse oximetry, capnography and direct radial artery pressures. All patients were kept nornothermic with a warming blanket. Normotensive anesthesia was maintained throughout the operation.

After approximately 45 minutes post-induction, a train of 4-twitch assessment was performed using a nerve stimulator (Fischer Paykel NS242, United Kingdom) on the median nerve over the wrist. Cortical stimulation was commenced only when the amplitude of the fourth twitch (abductor pollicis muscle) was visibly similar to the first, suggesting that the effects of neuromuscular blocking agents have subsided. An interval of 1 to 2 minutes was allowed between each train of cortical stimulation.

MEPs from the TA muscles were recorded bilaterally from the lower limbs. Peak to peak amplitudes (between 2 largest peaks opposite in polarity) and onset latency was measured for MEP responses in each limb, obtained from ipsilateral and contralateral cortical stimulation. Hence, ipsilateral MEPs refer to MEPs recorded from the TA on the same side as cortical stimulation. For each patient, 10 consecutive supramaximal MEPs obtained before insertion of pedicle screws were averaged to obtain a final mean amplitude and latency as a baseline. In addition, we also determined the first ('threshold') MEP response and initial stimulating intensity, defined as the mean of minimal intensities required to obtain 5 consistent MEP responses of at least 20 μV. In some circumstances, the first MEPs to appear were > 20 μV; below this corresponding stimulating intensity, no MEPs were elicited.

During insertion of pedicle screws and instrumentation, a 50% reduction of the MEP amplitude or 10% prolongation of latency was brought to the surgeon's attention, but active intervention was left to the surgeon's discretion. The surgical decision made immediately usually entails removal of pedicle screws, loosening or removal of correction rods.

The Wilcoxon Signed Ranks and Mann-Whitney tests were employed for statistical comparisons, with significant difference defined as p < 0.05.

## Results

For first MEP responses, the cross scalp stimulating position resulted in MEPs that were of significantly higher amplitude (p = 0.02), shorter latency (p = 0.03) and required lower stimulating intensity (p = 0.001) overall to elicit, as compared to the midline stimulating position.

For maximum MEP responses, similar findings were noted. The cross scalp stimulating position resulted in supramaximal MEPs that were of significantly higher amplitude (p = 0.001), shorter latency (p = 0.03) and required lower stimulating intensity (p = 0.001) to elicit overall, as compared to the midline stimulating position.

For first MEP responses, however, no significant differences were noted between ipsilateral and contralateral MEPs in terms of amplitude (p = 0.4), latency (p = 0.6) and intensity (p = 0.8). This was evident regardless of side of stimulation for both stimulating positions (p > 0.05 for all).

For maximum MEP responses, similarly, no significant differences were noted between ipsilateral and contralateral MEPs in terms of amplitude (p = 0.8), latency (p = 0.8) and intensity (p = 0.3). This was evident regardless of side of stimulation for both stimulating positions (p > 0.05 for all).

Comparisons between first and maximum MEP responses were made for amplitude, latency and stimulating intensity. For amplitude, a significant difference was observed with the cross scalp stimulating position (p = 0.001), with the maximum MEP responses showing larger amplitudes compared to the first MEP responses. This was not evident with the midline stimulating position (p = 0.4). Both maximum ipsilateral (p = 0.02) and maximum contralateral MEPs (p = 0.007) showed higher amplitudes than first MEP responses. For stimulating intensity, only the cross scalp stimulating position required a significantly larger intensity to achieve maximum MEP response amplitudes (p = 0.02). No significantly larger stimulating intensities were required to achieve ipsilateral (p = 0.23) vs. contralateral MEP (p = 0.18) responses. In terms of latencies, no significant differences were observed comparing between the first and maximum MEP responses elicited with both midline (p = 0.9) or cross scalp (p = 0.9) stimulating positions. Similarly, there were no significant latency differences comparing between the first and maximum ipsilateral (p = 0.87) and contralateral (p = 0.97) MEP responses. There were no MEP changes recorded among the 14 patients that required the surgeon to stop the operation to address the change in MEP.

**Figure 1:**
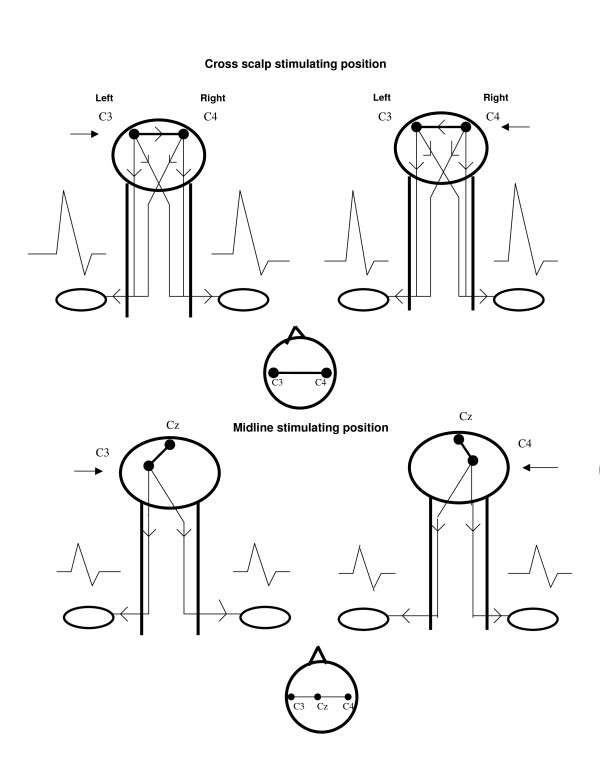
**Diagrammatic representation depicting summation of ipsilaterally and contralaterally generated MEPs from cortical stimulation, as a result of transcallosal or deep white matter conduction of stimulating current in the cross scalp stimulating position (top row)**. This has resulted in MEPs of larger amplitudes than those obtained with the midline stimulating position (bottom row). Arrows indicate the site of active cortical stimulating electrode.

**Figure 2:**
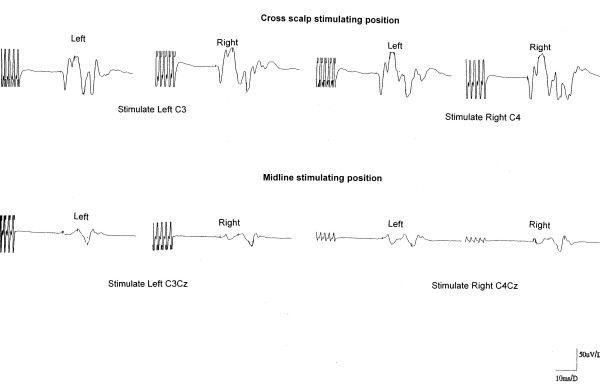
**Actual MEP traces of a representative patient corresponding to stimulating protocol depicted in Figure 1**. Latencies and amplitudes of MEPs are in reference to scale provided in the bottom right of the illustration.

Tables 1 and 2 summarize MEP data of the study. Figure 1 shows a diagrammatic representation of our findings. Figure 2 depicts actual MEP tracings of a patient.

**Table 1:** Comparison of MEP parameters obtained with cross scalp and midline stimulating positions

Parameter	Latency (ms)	Amplitude (mV)	Intensity (mA)
First MEP			
Cross scalp	27.9 (2.7)	71.3 (37.3)	52.9 (15.5)
Midline	29.2 (3.7)	43.5 (38.1)	76.4 (18.3)
			
Maximum MEP			
Cross scalp	27.7 (2.8)	130.5 (45.1)	61.8 (13.7)
Midline	29.1 (3.8)	57.9 (46.5)	80.5 (15.4)

Each value indicates mean and (standard deviation)

**Table 2:** Comparison of ipsilateral and contralateral MEP parameters

Parameter	Latency (ms)	Amplitude (mV)	Intensity (mA)
First MEP			
Ipsilateral MEP	28.6 (3.1)	59.9 (37.9)	64.7 (15.1)
Contralateral MEP	28.5 (3.2)	54.8 (29.2)	64.6 (16.2)
			
Maximum MEP			
Ipsilateral MEP	28.4 (3.2)	94.6 (39.4)	70.6 (14.1)
Contralateral MEP	28.4 (3.2)	93.8 (41.6)	71.7 (13.5)

Each value indicates mean and (standard deviation)

## Discussion

The main findings of our study were that the cross scalp stimulating position resulted in MEPs that were of significantly higher amplitude, shorter latency and required lower stimulating intensity overall to elicit, as compared to the midline stimulating position. This was evident for both first and maximum MEP responses. Thus, the cross scalp stimulating position was more effective overall in obtaining MEP responses during IOM of scoliosis surgery. The larger MEPs obtained may be the result of summation of ipsilateral and contralateral MEPs obtained bilaterally.

Additionally, both stimulating positions elicited ipsilateral and contralateral MEPs with no significant differences in terms of amplitude, latency and stimulation intensity individually. This was similarly observed for both first and maximum MEP responses. This finding suggests that ipsilateral and crossed contralateral motor tracts were activated effectively with both stimulating positions.

The comparisons of first and maximum MEP responses obtained also provide additional information regarding cortical stimulation. Significantly larger maximum MEP amplitudes compared to first MEP responses were obtained only with cross scalp stimulation. Moreover, only the cross scalp stimulating position required a significantly larger intensity to achieve maximum MEP response amplitudes. These findings suggest that this position likely achieved a more distributed and possibly deeper stimulating current across the scalp, as opposed to the more focal midline stimulating position. Finally, latency differences were not demonstrated between the first and maximum MEP responses with both stimulating positions individually, suggesting that early and late MEP components were effectively obtained overall, even with the initial MEPs obtained with lower stimulating intensities during the time when maximum MEP amplitudes were not elicited.

In summary, cross scalp stimulation achieved effective bilateral, symmetrical motor cortex activation, likely with more distributed current passage into the motor areas. It is likely that transmission of stimulating currents across both motor cortices had resulted in these observations. The midline stimulating position also achieved effective MEP elicitation. However, the smaller MEP amplitudes obtained, coupled with longer latencies with the midline position, suggest more focal, unilateral motor cortex activation. Specifically, the longer latencies obtained with the midline stimulating position may be due to ineffective stimulation of ipsilaterally conducted MEPs. These ipsilateral MEPs have shorter latencies and are summated early with contralateral MEPs to manifest as larger and earlier occurring MEPs seen with the cross scalp stimulating position.

Unilateral cortical electrical stimulation at the fronto-central region was previously described using a frontal anode, in conjunction with a medially placed cathode [3]. This was in contrast to our method, which employed an active stimulating electrode, in conjunction with an anode medially at Cz. Transcallosal conduction of electrical stimulation has been well described in cat experiments, whereby a small increase in stimulating current resulted in bilateral cortical activation, likely propagated via the rostral corpus callosum [4]. These observations corroborate our findings obtained in the human brain.

What are the physiological correlates of our findings? Our observations have pointed to transcallosal stimulation of the contralateral motor cortex in the cross scalp stimulating position. There exist several lines of evidence to support this. Physiologically, rat brain studies have demonstrated widespread action of anesthesia at multiple binding sites, and the effects of anesthesia on corticospinal excitability may facilitate our MEP observations [5]. Structurally, magnetic resonance brain imaging has also demonstrated increased callosal T2 changes with anesthesia, suggesting morphological alterations at the tissue level [6]. Functionally, it is also possible that longstanding scoliosis has led to spinal cord plasticity changes. Motor pathway reorganization and spinal cord plasticity have been well documented in response to cord injury [7] in an activity-dependent manner [8]. Thus, structural and postural changes of longstanding scoliosis may have resulted in reorganization of cortical or subcortical motor pathways, including ipsilateral corticoreticular fibres leading to our MEP observations after cortical stimulation[9]. In all, the corpus callosum, which is a large white matter tract playing a vital role in interhemispheric interactions, is likely to be crucially engaged in our observations intraoperatively [10].

A previous study by Szelenyi et al [11] can be best compared to ours. The former had postulated that stimulating of deep white matter motor tracts may be suggested by simultaneous recording of ipsilateral and contralateral MEPs, or with higher stimulation intensity. This is corroborated by our findings with cross scalp stimulation. Hence, in addition to the possibility of transcallosal conduction, deep white matter stimulation can be an additional explanation for our findings. To this end, the more focal midline stimulation would be suitable for supratentorial neurosurgical resection procedures. In contrast, the exact site of stimulation is of less importance for spinal surgery which takes place at a more caudal location [11].

During IOM for procedures carrying real risks of spinal cord injury, the descending corticospinal tracts should be activated as fully as possible. We had previously shown that concurrent ipsilateral and contralateral MEP amplitude changes obtained with cortical stimulation were of value in reducing false positive observations during IOM [12]. While we feel that any reduction above 50% in MEP amplitude (rather than complete disappearance) warrants alerting the surgical team by erring on the side of caution, this may occasionally result in false positive outcomes. However, concurrent ipsilateral and contralateral MEP amplitude changes point to a definite surgical urgency. In this situation, ipsilateral MEPs are of value in reducing false positive observations during IOM. The smaller amplitude MEPs obtained with the midline stimulating position suggests that this method may not be adequate under operative circumstances where anesthetic agents may cause suppression of cortical and spinal excitability [13,14]. For these reasons, we advocate the use of the cross scalp stimulating position for IOM of spinal surgery.

## Competing interests

The authors declare that they have no competing interests.

## Authors' contributions

YLL conceptualized and wrote the paper. YFD, YET and AT helped with the technical aspects. SBT, WMY and CMG helped with the concepts and surgery. SF-C helped with manuscript preparation and statistical inputs. All authors read and approved the final manuscript.
